# Supplementary material for: Comparison of colorimetric, fluorometric, and liquid chromatography-mass spectrometry assays for acetyl-coenzyme A
Source: bioRxiv. 2023 Jun 1:2023.06.01.543311. Preprint. [Version 1] doi: 10.1101/2023.06.01.543311 (PMC10312605; doi:10.1101/2023.06.01.543311)
Supplement: Supplementary file 1 [file NIHPP2023.06.01.543311v1-supplement-1.pdf]

bioRxiv preprint doi: https://doi.org/10.1101/2023.06.01.543311; this version posted June 1, 2023. The copyright holder for this preprint (which was not certified by peer review) is the author/funder, who has granted bioRxiv a license to display the preprint in perpetuity. It is made available under aCC-BY 4.0 International license.

Figures (in order of appearance)

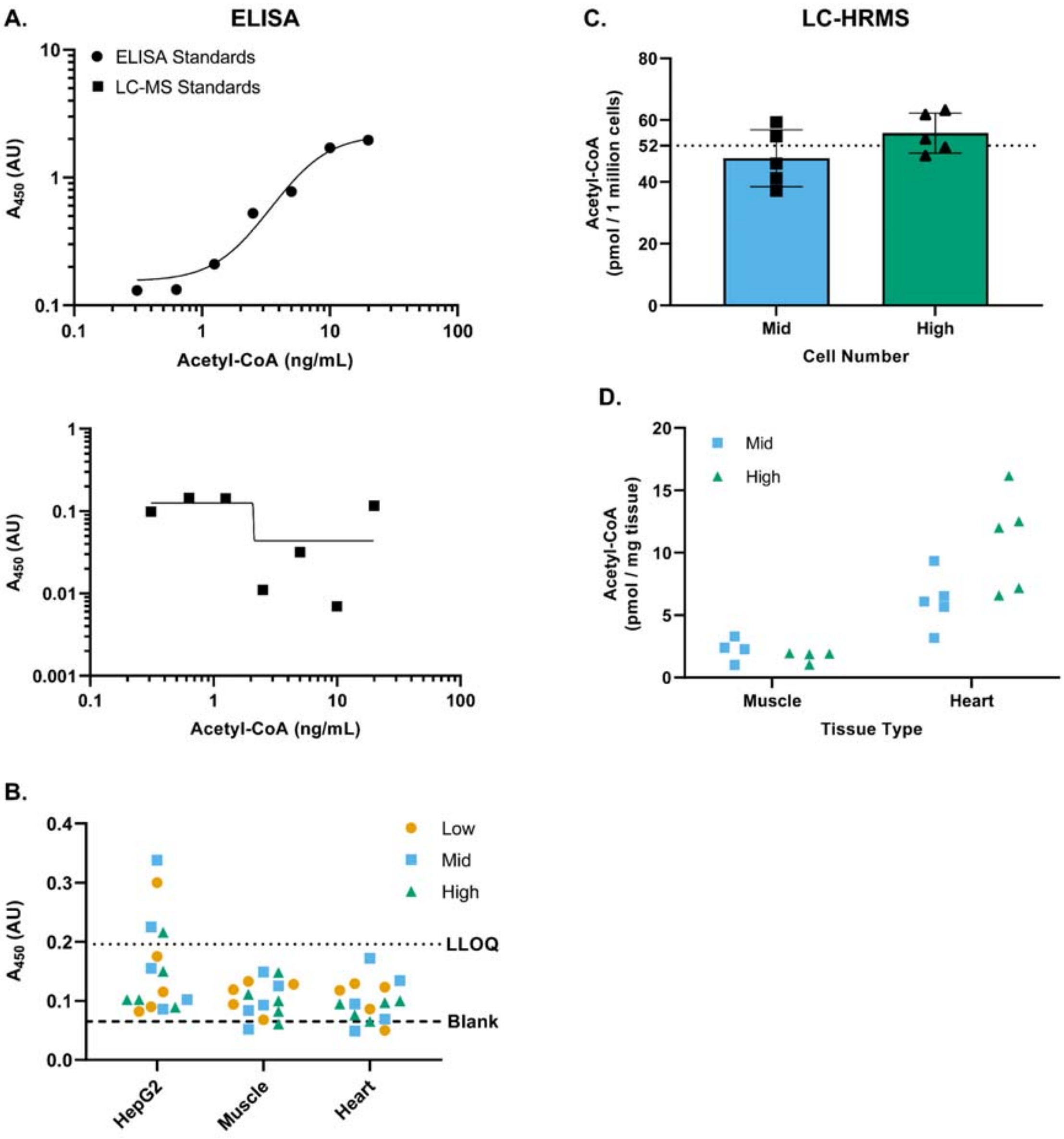

Supplemental Figure 1. Acetyl-CoA ELISA kit does not provide reliable quantitation of acetyl-CoA in cell or tissue samples.

Acetyl-CoA was measured in HepG2 cells and tissue samples (mouse skeletal muscle and heart) via an Elabscience acetyl-CoA ELISA kit or via LC-HRMS. For cell samples, 100 µL of 1-mL metabolite extracts from 10 million (high), 1 million (mid), or 100,000 (low) cells was analyzed. For tissue samples, 100 µL of 1-mL extracts of ~30 mg (high), ~10 mg (mid), or a 1:10 dilution of the 10 mg extract (low) was analyzed.

(A) Four-parameter logistic curves generated via ELISA using standards prepared from Elabscience ELISA (top) or LC-MS (Sigma-Aldrich) (bottom) acetyl-CoA stock standard solutions. Absorbance from the blank was subtracted from the absorbance of each standard prior to plotting per the protocol.

(B) Raw absorbance values of cell and tissue samples analyzed via ELISA. The lower limit of quantitation (LLOQ) (dotted line) refers to the raw absorbance of the lowest concentration

bioRxiv preprint doi: https://doi.org/10.1101/2023.06.01.543311; this version posted June 1, 2023. The copyright holder for this preprint (which was not certified by peer review) is the author/funder, who has granted bioRxiv a license to display the preprint in perpetuity. It is made available under aCC-BY 4.0 International license.

standard (0.04 pmol/well), and the Blank (dashed line) refers to the raw absorbance of the blank, which did not contain added acetyl-CoA. Note that only a few cell samples exhibited a raw absorbance above that of the LLOQ absorbance, and the response did not scale with the number of cells utilized.

(C) Acetyl-CoA measured in HepG2 cells via LC-HRMS. Each symbol represents an individual replicate sample, and error bars represent standard deviations. The overall mean (n=10) of the mid and high samples was 52 pmol/million cells. The low samples exhibited a response below the lower limit of quantitation.

(D) Acetyl-CoA measured in mouse skeletal muscle and heart tissue via LC-HRMS. Each symbol represents tissue from one of four (skeletal muscle) or five (heart) different mice. The majority of the low samples exhibited a response below the lower limit of quantitation and, therefore, are not shown.

bioRxiv preprint doi: https://doi.org/10.1101/2023.06.01.543311; this version posted June 1, 2023. The copyright holder for this preprint (which was not certified by peer review) is the author/funder, who has granted bioRxiv a license to display the preprint in perpetuity. It is made available under aCC-BY 4.0 International license.

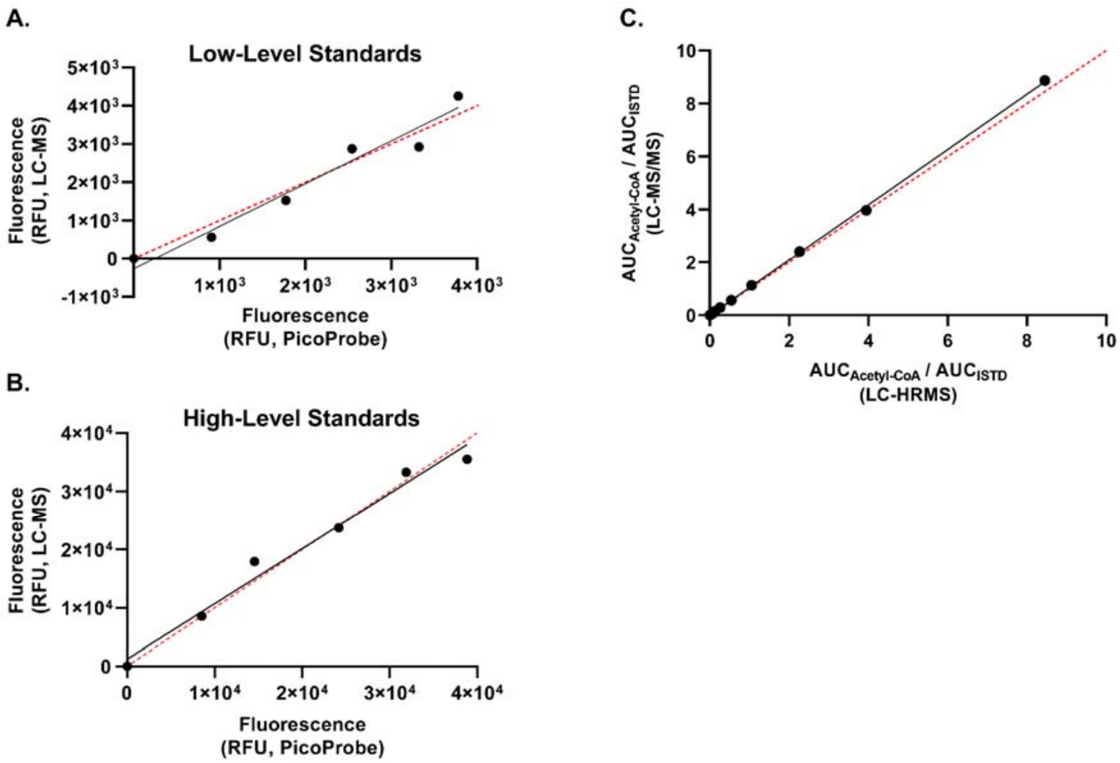

Supplemental Figure 2. Deming regression analysis of PicoProbe™ and LC-MS standards analyzed via the fluorometric PicoProbe™ kit.

Deming regression analysis was performed for (A) low- and (B) high-level standard curves generated using the PicoProbe™ assay kit. The PicoProbe™ assay standards were prepared per the manufacturer's recommendations. A set of standards also was prepared at the same concentrations using the stock standard used for the LC-MS assays. The dashed line represents the y=x line of agreement. RFU = relative fluorescence units

(C) Deming regression analysis was performed for standard curves generated via LC-HRMS and LC-MS/MS assays. The dashed line represents the y=x line of agreement. AUC = area under the curve

bioRxiv preprint doi: https://doi.org/10.1101/2023.06.01.543311; this version posted June 1, 2023. The copyright holder for this preprint (which was not certified by peer review) is the author/funder, who has granted bioRxiv a license to display the preprint in perpetuity. It is made available under aCC-BY 4.0 International license.

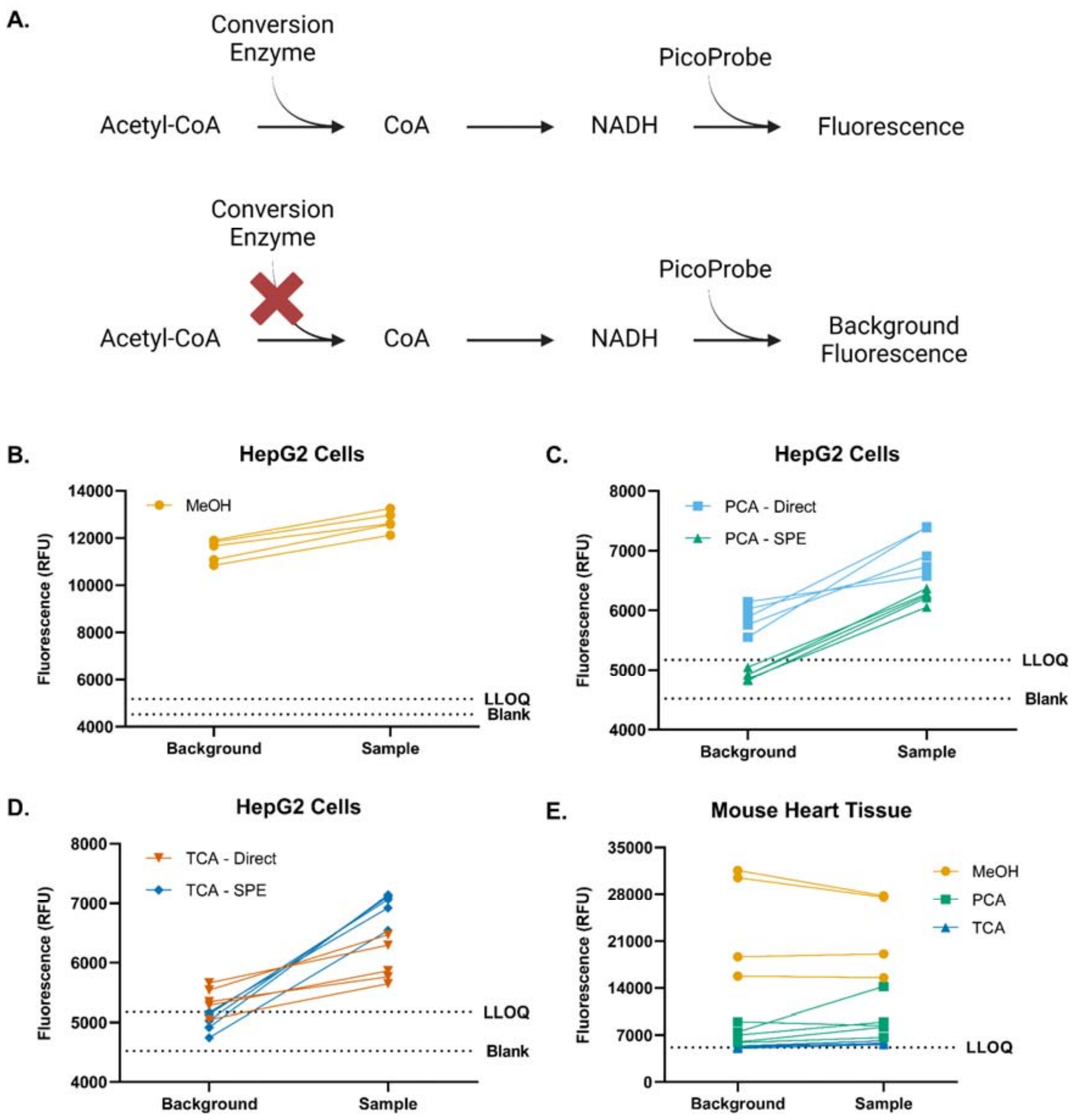

Supplemental Figure 3. High background fluorescence interferes with PicoProbe™ measurements in mouse heart tissue samples.
(A) Schematic of how background sample fluorescence is determined via the PicoProbe™ assay. The sample is added in equal volumes to each of two wells of a 96-well plate. To one well, a reaction mix containing the conversion enzyme is added. To the other well, a reaction mix omitting the conversion enzyme is added to evaluate background sample fluorescence.
(B-D) Acetyl-CoA was extracted from HepG2 cells using (B) -80°C 80:20 methanol:water (MeOH), (C) perchloric acid (PCA) or (D) 10% trichloroacetic acid in water (TCA) as described in the methods section and then analyzed via the PicoProbe™ assay. Raw fluorescence from the background correction samples and the test samples was plotted, with lines connecting the corresponding background and test samples.
(E) Acetyl-CoA was extracted from mouse heart tissue using MeOH, PCA, or TCA as described in the methods section and then analyzed via the PicoProbe™ assay. Raw fluorescence from the background correction samples and the test samples was plotted as in (B-D).

bioRxiv preprint doi: https://doi.org/10.1101/2023.06.01.543311; this version posted June 1, 2023. The copyright holder for this preprint (which was not certified by peer review) is the author/funder, who has granted bioRxiv a license to display the preprint in perpetuity. It is made available under aCC-BY 4.0 International license.

Each symbol represents an individual replicate sample.

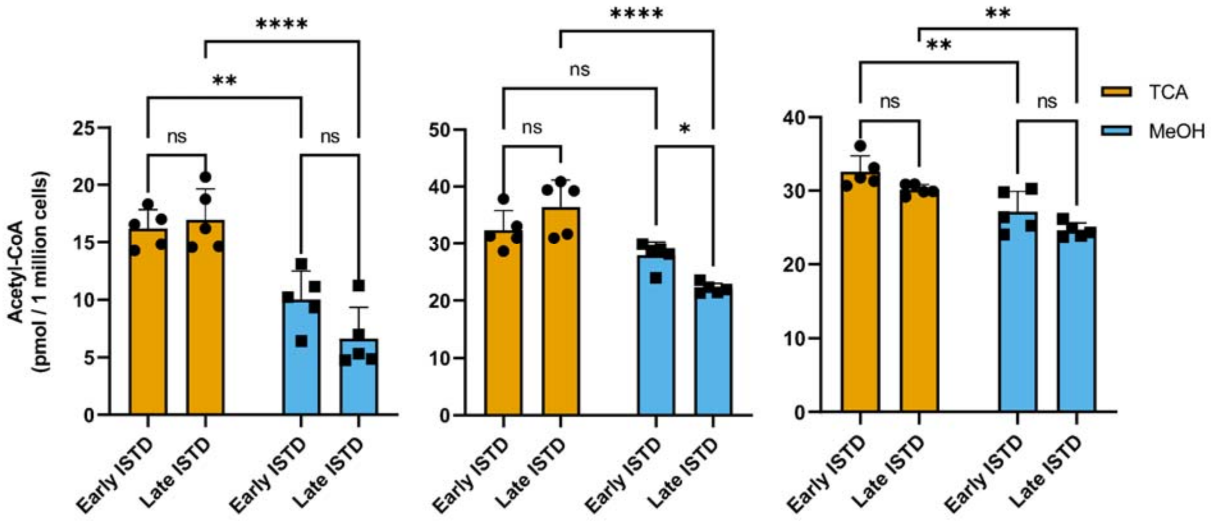

Supplemental Figure 4. Acetyl-CoA concentration measured via LC-HRMS is affected by extraction method.
Acetyl-CoA was extracted from HepG2 cells using -80°C 80:20 methanol:water (MeOH) or 10% trichloroacetic acid in water (TCA) as described in the methods section and analyzed via LC-HRMS. During sample processing, the internal standard was added either prior to sonication (“early ISTD”) or after sonication (“late ISTD”). Each symbol represents an individual replicate sample, and error bars represent standard deviations. Each graph represents an individual experiment. Statistical comparisons were performed via two-way ANOVA with post-hoc Tukey’s correction for multiple comparisons. *p≤0.05, **p≤0.01, ****p≤0.0001, ns = no significance

bioRxiv preprint doi: https://doi.org/10.1101/2023.06.01.543311; this version posted June 1, 2023. The copyright holder for this preprint (which was not certified by peer review) is the author/funder, who has granted bioRxiv a license to display the preprint in perpetuity. It is made available under aCC-BY 4.0 International license.

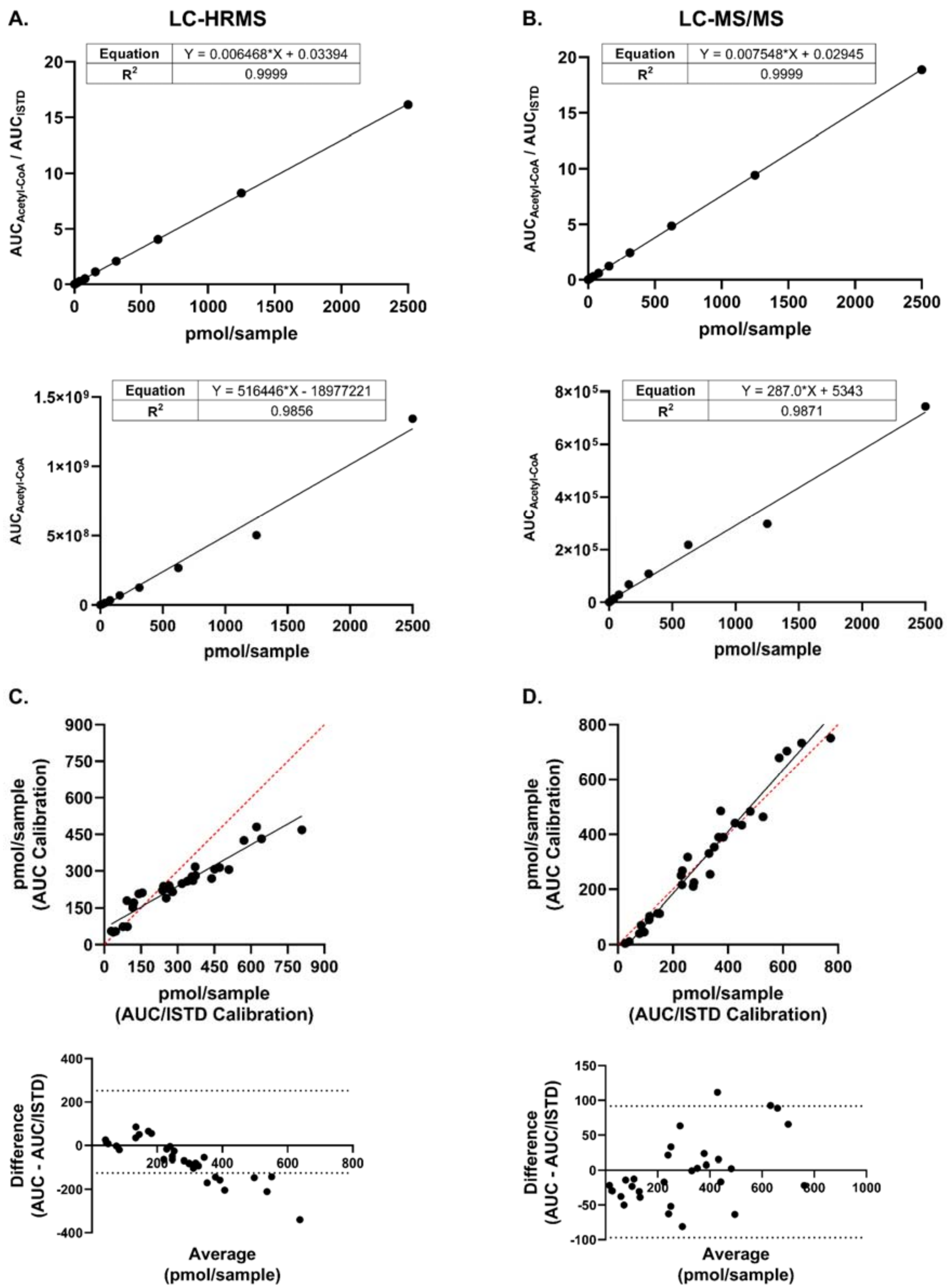

Supplemental Figure 5. Using an internal standard for LC-MS analysis improves linearity
(A and B) Callibration curves were generated via (A) LC-HRMS or (B) LC-MS/MS analysis using either the ratio of the acetyl-CoA peak area (AUC) and the internal standard peak area (top) or only the acetyl-CoA peak area (bottom).

bioRxiv preprint doi: https://doi.org/10.1101/2023.06.01.543311; this version posted June 1, 2023. The copyright holder for this preprint (which was not certified by peer review) is the author/funder, who has granted bioRxiv a license to display the preprint in perpetuity. It is made available under aCC-BY 4.0 International license.

(C and D) Deming regressions (top) and Bland-Altman plots (bottom) were generated from the sample results interpolated from the two different standard curves measured via (A) LC-HRMS or (B) LC-MS/MS analysis. The dashed line represents the y=x line of agreement.
Data is representative of n=2 independent experiments.

bioRxiv preprint doi: https://doi.org/10.1101/2023.06.01.543311; this version posted June 1, 2023. The copyright holder for this preprint (which was not certified by peer review) is the author/funder, who has granted bioRxiv a license to display the preprint in perpetuity. It is made available under aCC-BY 4.0 International license.

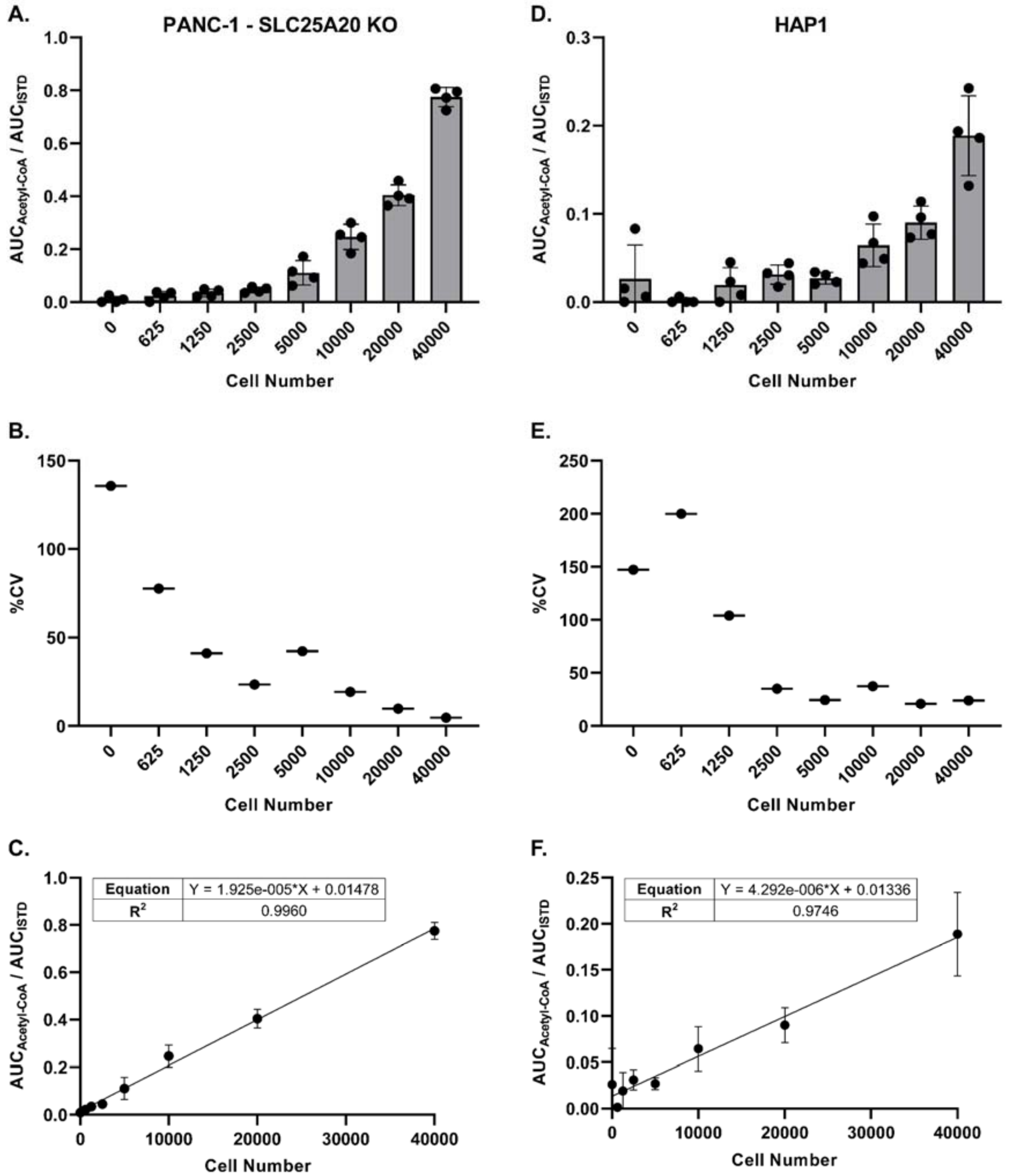

Supplementary Figure 6. The ability of LC-HRMS analysis to achieve precise relative quantitation of acetyl-CoA at low cell numbers is cell-line dependent.
Panc-1 SLC25A20 or HAP1 cells were plated in a 96-well plate at various cell numbers (n=4 wells per cell number), incubated overnight, and processed directly for LC-HRMS analysis.
(A and D) Ratio of the peak area (AUC) of acetyl-CoA versus the peak area of the acetyl-CoA internal standard. Each symbol represents an individual replicate, and error bars represent standard deviations.
(B and E) Percent coefficient of variation of the individual replicates (n=4) at each cell number. Some replicates at the lower cell numbers did not have a detectable acetyl-CoA response.
(C and F) Linear regression of the peak area ratios. Each symbol represents the mean of individual replicates (n=4). Error bars represent standard deviations.
